# Quantitative and molecular adjudication of BioFire FilmArray and QIAstat-Dx discordance in meningitis/encephalitis diagnostics

**DOI:** 10.1128/spectrum.01333-26

**Published:** 2026-09-02

**Authors:** Rainer Gosert, Christopher M. Field, Roger Koller, Dominic C. Mills, Klaudia Naegele, Maximilian Koester, Jakob Meyer, Ulrich Heininger, Tobias Derfuss, Lena Jaki, Stefano Bassetti, Reto Lienhard, Antony Croxatto, Roland Bingisser, Christian H. Nickel, Pascal Bittel, Guido Bloemberg, Sarah Tschudin-Sutter, Nina Khanna, Peter M. Keller, Karoline Leuzinger

**Affiliations:** 1 Clinical Virology, University Hospital Basel 30262 , Basel, Switzerland; 2 Laboratory Medicine, University Hospital Basel 30262 , Basel, Switzerland; 3 Institute for Infectious Diseases, University of Bern 27210, Bern, Switzerland; 4 Institute of Medical Virology, University of Zürich 30988, Zürich, Switzerland; 5 Clinical Bacteriology/Mycology, University Hospital Basel 30262 , Basel, Switzerland; 6 Emergency Medicine, University Hospital Basel 30262 , Basel, Switzerland; 7 Pediatric Infectious Diseases and Vaccinology, University of Basel Children’s Hospital 572245, Basel, Switzerland; 8 Neurology, University Hospital Basel 30262 , Basel, Switzerland; 9 Internal Medicine, University Hospital Basel 30262 , Basel, Switzerland; 10 ADMED Microbiology 211140 , La Chaux-de-Fonds, Switzerland; 11 Division of Infectious Diseases, University Hospital Basel 30262 , Basel, Switzerland; 12 Department of Biomedicine, University of Basel 89390, Basel, Switzerland; Sacramento County Public Health Laboratory, Sacramento, California, USA

**Keywords:** cerebrospinal fluid, syndromic panel testing, meningitis/encephalitis panel, enterovirus, FilmArray, QIAstat-Dx

## Abstract

Syndromic multiplex panels enable rapid cerebrospinal fluid (CSF) diagnosis of meningitis and encephalitis, but clinically relevant inter-platform discordance persists, and its analytical basis remains incompletely understood. In this retrospective multicenter study, 182 residual CSF specimens from 182 patients at 4 Swiss tertiary-care centers and 10 external quality assessment samples were tested centrally by the QIAstat-Dx Meningitis/Encephalitis panel and compared with routine BioFire FilmArray results. Discordant results were adjudicated using pathogen-specific quantitative laboratory-developed tests (LDTs) calibrated to external standards, and enterovirus-positive specimens underwent molecular typing. Among 182 clinical CSF specimens, concordance was 87.9% across shared targets. Complete agreement was observed for herpes simplex virus type 2, *Haemophilus influenzae*, *Neisseria meningitidis*, *Streptococcus agalactiae*, and *Cryptococcus neoformans/gattii*. Across the remaining shared targets, positive percent agreement ranged from 50% to 93% and negative percent agreement from 98% to 100%. Enterovirus showed the greatest discordance, with 86% positive percent agreement and 10 FilmArray-only detections. LDT testing confirmed low-level enterovirus positivity in four discordant specimens (100‒700 copies/mL), and discordant viral detections clustered at lower target burden than concordant positives (*P* < 0.001). Molecular typing identified enterovirus species A, B, and C in both concordant and FilmArray-only positive specimens. In conclusion, FilmArray and QIAstat-Dx showed high agreement for CSF pathogen detection. Residual discordance was largely confined to low-burden viral detections, particularly enterovirus, and was more consistent with near-threshold target abundance than with a major type-restricted detection gap. These findings support routine syndromic CSF testing and emphasize interpretation of borderline positives in conjunction with confirmatory LDT testing and clinical context.

**IMPORTANCE:**

Syndromic cerebrospinal fluid panels are increasingly used as first-line tests for suspected central nervous system infection, yet aggregate agreement metrics can obscure clinically relevant differences near the limit of detection. In this multicenter comparison, most discordance clustered in low-burden enterovirus-positive cerebrospinal fluid (CSF) specimens and showed no clear segregation by enterovirus type, indicating that disagreement largely arose from near-threshold detection rather than a major gap in assay inclusivity. The broader implication is that performance comparisons of syndromic panels should move beyond overall agreement and specifically address how platforms handle borderline-positive results. This matters clinically because low-level viral detections can alter post-test probability, antimicrobial stewardship, CSF resampling, and follow-up microbiologic workup. More generally, the data support laboratory-integrated diagnostic strategies that combine multiplex screening with quantitative adjudication, molecular characterization, and interpretation of semiquantitative signals to strengthen the analytical and clinical utility of syndromic testing for central nervous system infections.

## INTRODUCTION

Acute infectious meningitis and encephalitis (ME) are neurological emergencies in which rapid etiological clarification is essential to guide targeted antimicrobial therapy and timely clinical management (1). Although bacterial meningitis remains associated with high morbidity and mortality, viruses represent the most frequent cause of community-acquired ME in routine clinical practice, with herpes simplex virus type 1 (HSV-1), herpes simplex virus type 2 (HSV-2), varicella-zoster virus (VZV), and enteroviruses (EV) accounting for most laboratory-confirmed cases in cerebrospinal fluid (CSF) by nucleic acid testing (2, 3). Syndromic multiplex panel testing has substantially improved the speed and breadth of CSF pathogen detection; however, inter-platform differences remain a relevant limitation, particularly for low-level viral positives near the limit of detection, where discordant results occur most frequently and may complicate diagnostic interpretation.

The BioFire FilmArray ME panel (bioMérieux), introduced in 2016, was the first syndromic multiplex panel test designed for ME diagnostics and allows simultaneous detection of 14 bacterial, viral, and yeast targets from CSF within approximately 1 h (4). More recently, the QIAstat-Dx ME Panel (Qiagen) became available as a second cartridge-based syndromic platform with largely overlapping pathogen coverage, while differing in selected targets, including the addition of *Streptococcus pyogenes* and *Mycoplasma pneumoniae* and the absence of cytomegalovirus (CMV) in version 1.1 (Table S1). In contrast to the qualitative FilmArray readout, the QIAstat-Dx system also provides cycle threshold (Ct) values and amplification curves, which may offer additional information on pathogen burden and analytical confidence in low-copy-number specimens (1, 5, 6). Recent comparative studies have shown high overall concordance between the two platforms, with positive percent agreement (PPA) and negative percent agreement (NPA) generally exceeding 95% (1, 5, 6). However, these studies relied mainly on aggregate agreement measures and therefore provided limited insight into discordant viral detections near the lower end of analytical sensitivity. In particular, it remains unresolved whether such discordances represent false positive signals, false negative results on the comparator platform, or borderline true positives close to the limit of detection. This question is especially relevant for EV, which are among the most frequently detected viral pathogens in CSF, are genetically highly diverse, and often occur at low copy number (7). EV therefore provides an informative model to assess whether inter-platform discordance is primarily related to low viral burden or may also be influenced by EV-type diversity, including uncommon or potentially neurotropic types (8).

To address these gaps, this study retrospectively compared the QIAstat-Dx ME panel and the BioFire FilmArray ME panel in a multicenter set of native CSF specimens and adjudicated discordant results using pathogen-specific laboratory-developed tests (LDTs) calibrated to external standards. Enterovirus-positive specimens were further characterized by molecular typing to assess whether discordance was driven primarily by low target burden or by gaps in detection across enterovirus diversity.

## MATERIALS AND METHODS

### Study design and specimen collection

This retrospective multicenter comparative study included 182 residual clinical CSF specimens from 182 patients and 10 external quality assessment (EQA) specimens. Clinical specimens were obtained during routine diagnostic work-up of patients with suspected meningitis or encephalitis at the University Hospital Basel, the University Hospital Bern, the Regional Hospital Neuchatel, and the University Hospital Zurich between 2022 and 2024 (Fig. S1). Initial testing was performed locally at the participating centers using the BioFire FilmArray ME panel. Residual CSF specimens were subsequently forwarded to the Clinical Virology Unit at the University Hospital Basel for centralized retrospective testing with the QIAstat-Dx ME panel. Because participating centers preferentially submitted specimens with positive local BioFire FilmArray ME results, the clinical study set was non-consecutive and enriched for positive targets. Accordingly, the study was designed to assess inter-platform agreement rather than pathogen prevalence, routine positivity rates, or diagnostic yield in an unselected patient population. Patient demographics are summarized in Table S2. EQA specimens, consisting of materials with predefined positive targets, were analyzed separately to increase representation of infrequently encountered pathogens.

### Dilution experiments for select viral targets

To compare detection of selected viral targets at low concentrations, negative CSF specimens were spiked with high-titer cell-culture supernatants containing whole virus particles of HSV-1, HSV-2, and different EV-types, including echovirus 9 and coxsackieviruses B1 and B6. The resulting high-titer CSF stocks were serially diluted in negative CSF to nominal target concentrations of 100, 1,000, and 10,000 viral genome copies per mL. Viral nucleic-acid concentrations in the stock preparations and dilution series were verified using the corresponding in-house quantitative LDT calibrated to external plasmid standards, as described below. Each dilution was tested in triplicate on the FilmArray and QIAstat-Dx panels to determine the lowest concentration at which each assay reproducibly yielded a positive target detection.

### Syndromic multiplex panel testing

Routine diagnostic testing was performed with the BioFire FilmArray ME panel (BioFire Diagnostics, UT, USA), and residual specimens were retrospectively tested with the QIAstat-Dx ME panel (Qiagen, Hilden, Germany) according to the manufacturers’ instructions.

For the FilmArray, 200 μL of CSF was mixed with the supplied sample buffer, loaded into the pouch, and processed on the FilmArray instrument, with a run time of approximately 65 min. For the QIAstat-Dx, 200 μL of CSF was loaded directly into the cartridge and processed on the QIAstat-Dx Analyzer 1.0, yielding results in approximately 80 min. The two panels showed broad target overlap. Both assays concurrently detected EV, HSV-1, HSV-2, human herpesvirus 6 (HHV-6), human parechovirus (HPeV), VZV, *Escherichia coli* K1, *Haemophilus influenzae, Listeria monocytogenes*, *Neisseria meningitidis*, *Streptococcus agalactiae*, *Streptococcus pneumoniae*, and *Cryptococcus neoformans/gattii*. The FilmArray ME panel uniquely included cytomegalovirus, whereas QIAstat-Dx ME panel version 1.1 uniquely included *Mycoplasma pneumoniae* and *Streptococcus pyogenes* (Table S1) (6). Regarding analytical readout, the FilmArray panel is intended as a qualitative assay and reports routine positive/negative results only. For exploratory analyses in this study, internal FilmArray crossing point (Cp) values were additionally extracted for positive targets. These values reflect the second-stage amplification cycle at which fluorescence exceeds a predefined threshold and are inversely related to the amount of target nucleic acid present in the specimen. In contrast, the QIAstat-Dx ME panel reports qualitative results together with Ct values and amplification curves, allowing exploratory comparison of low-level detections across platforms.

### Confirmatory testing and discrepancy resolution

Results obtained with FilmArray and QIAstat-Dx were compared for each shared target. Discordant results were adjudicated with target-specific LDTs using quantitative real-time PCR assays calibrated to external plasmid standards (Table S3). Quantification was performed using the plasmid standards, each containing the target genomic region of each pathogen. Plasmid standards were prepared at concentrations of 10^6^, 10^4^, and 10^2^ copies per mL and run in triplicate in each assay. These standards were used to generate calibration curves for quantification and to ensure assay performance consistency across runs. The lower limit of quantification (LLoQ) of the LDTs used for adjudication was 1,000 copies/mL. Target levels below this threshold were reported as detected/positive but were not considered reliably quantifiable. In adjudicated analyses, a discordant target was classified as confirmed positive when the corresponding LDT result was positive. The LDT was not treated as a gold standard, but as a supplementary method to verify positive findings from either evaluated panel. Because concordant-positive specimens were not systematically retested, these analyses are discrepancy-resolved rather than fully reference-standard-based estimates of diagnostic accuracy.

### Targeted next-generation sequencing and molecular typing of EV

Molecular typing of EV-positive specimens was performed according to published protocols (9), followed by targeted sequencing of the resulting amplicons on an Illumina MiniSeq platform (Illumina, CA, USA).

Sequencing reads were processed with BBDuk (BBTools v39.13) for adapter removal, PhiX filtering, and quality trimming (10). Adapter trimming was performed in right-trimming mode using a *k*-mer length decreasing from 23 to 11 with a Hamming distance of 1, followed by PhiX filtering with a *k*-mer length of 31 and a Hamming distance of 1. Low-quality bases were trimmed from both read ends using a Phred score cutoff of 14 and a minimum average quality score of 20. Reads shorter than 45 bp or containing ambiguous bases were discarded. For subtype assignment, a reference database was compiled from the NCBI nucleotide database on 21 January 2026 using the query txid12059[Organism:exp] AND “complete genome” and restricting sequence length to 1,000–10,000 bp. After exclusion of one non-genomic record, the final database comprised 8,618 EV reference sequences. Reads were competitively aligned against all references using BWA (v0.7.18) (11), and alignments were sorted and summarized with SAMtools (v1.21) (12). EV-type was assigned according to the reference sequence with the highest horizontal coverage.

### Statistical analyses

Demographic characteristics were summarized descriptively by age, sex, and predefined age groups. Inter-platform agreement across the 182 clinical CSF specimens was assessed at the target level, including overall agreement, PPA, NPA, and Cohen’s κ. For calculation of directional agreement metrics, FilmArray results were used as the denominator, but neither assay was treated as a reference standard. Statistical analyses were performed using R (https://www.r-project.org), and graphs were generated with GraphPad Prism (version 10; GraphPad Software, CA, USA).

## RESULTS

### Overall pathogen detection

Of 182 clinical CSF specimens, 156 (85.7%) were positive for at least one viral target by one or both syndromic panels. At the specimen level, 160/182 (87.9%) results were concordant, including 152 concordant-positive and 8 concordant-negative specimens (Table 1).

**TABLE 1:** Overall concordance of shared target detections between the BioFire FilmArray ME and QIAstat-Dx ME panels in clinical cerebrospinal fluid specimens

		QIAstat-Dx		Overall agreement	Cohen’s κ score
		Positive	Negative		
BioFire FilmArray	Positive	152	17	87.9%	0.36
	Negative	5	8		

**Fig 1:**
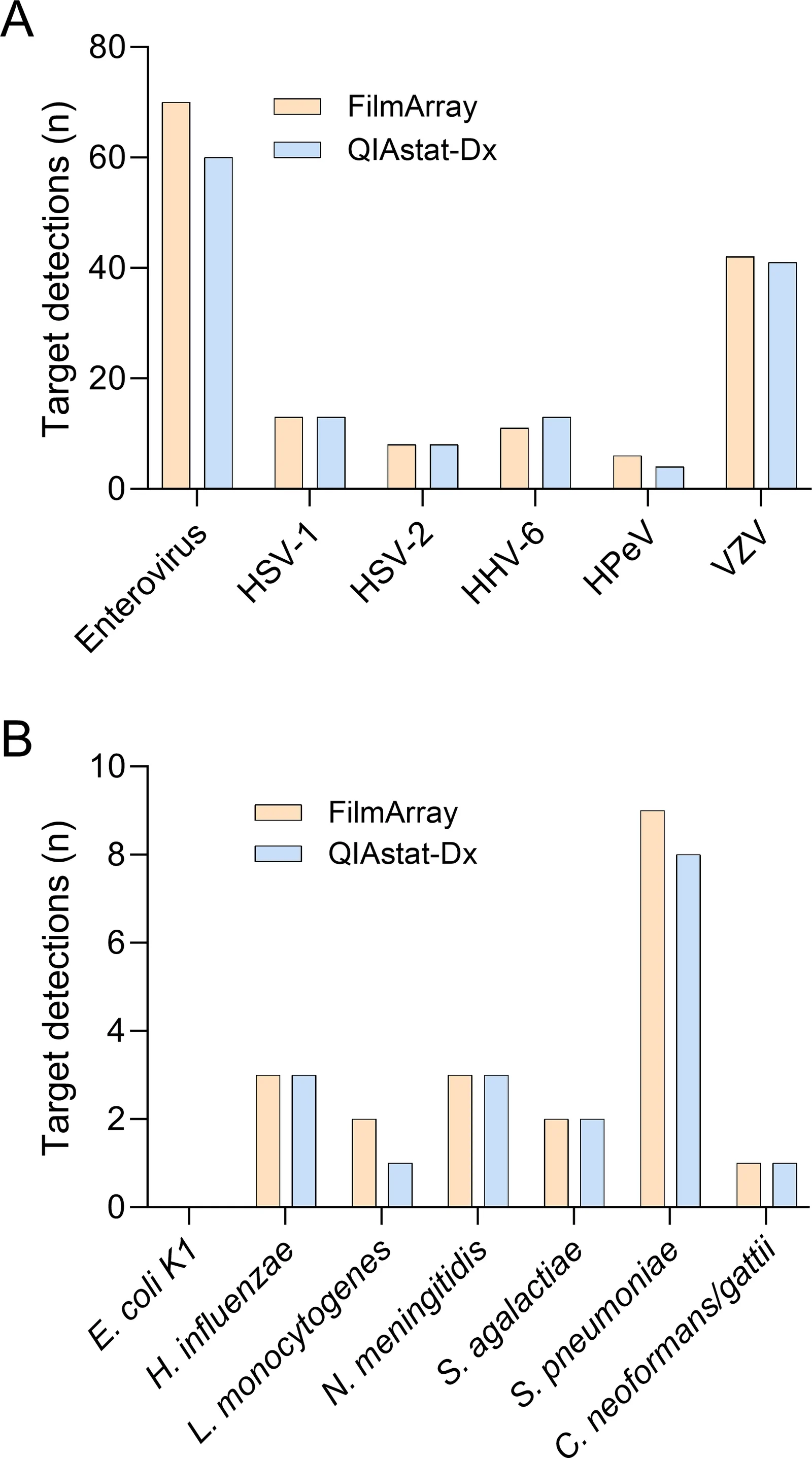
Detection of meningitis/encephalitis panel analytes in clinical cerebrospinal fluid specimens by syndromic multiplex panel testing. A total of 182 clinical cerebrospinal fluid specimens were tested with the BioFire FilmArray ME Panel and the QIAstat-Dx ME Panel. Bars show the number of detections by each platform for shared viral targets (**A**) and bacterial and fungal targets (**B**).

**Fig 2:**
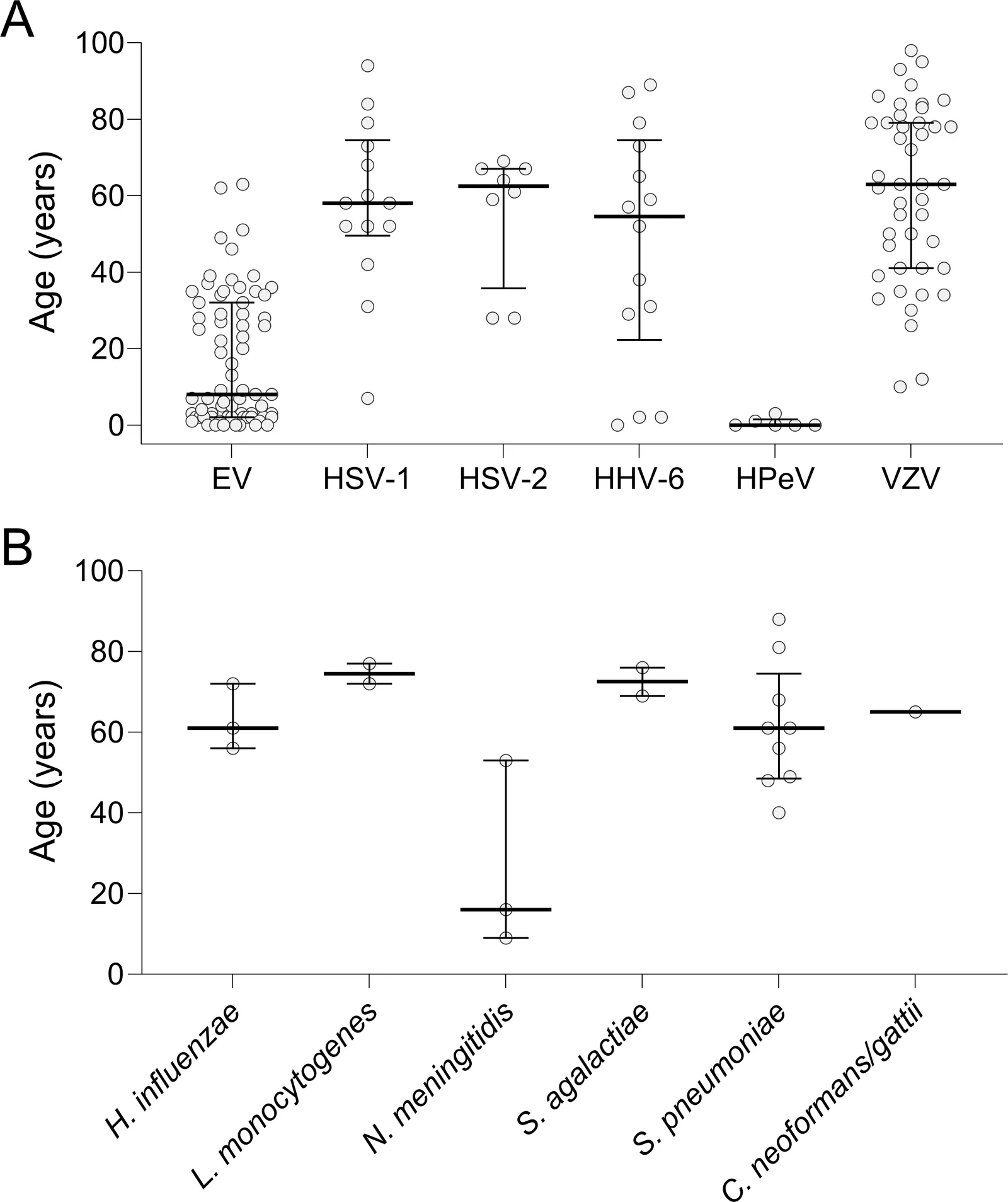
Age distribution of patients with positive meningitis/encephalitis panel analyte detections in clinical cerebrospinal fluid specimens. Each symbol represents the age of one patient with a positive detection of a viral target (**A**) or a bacterial or fungal target (**B**) in a clinical cerebrospinal fluid specimen. Horizontal bars indicate medians, and whiskers indicate the 25th and 75th percentiles. EV, enterovirus; HSV-1, herpes simplex virus 1; HSV-2, herpes simplex virus 2; HHV-6, human herpesvirus 6; HPeV, human parechovirus; VZV, varicella-zoster virus; *H. influenzae*, *Haemophilus influenzae*; *L. monocytogenes*, *Listeria monocytogenes*; *N. meningitidis*, *Neisseria meningitidis*; *S. agalactiae*, *Streptococcus agalactiae*; *S. pneumoniae*, *Streptococcus pneumoniae*; *C. neoformans/gattii*, *Cryptococcus neoformans/gattii*.

At the analyte level, EV (*n* = 70) and VZV (*n* = 44) were the most frequently detected viruses (Fig. 1A). Bacterial and fungal detections were less common and composed of nine *S. pneumoniae*, three *N. meningitidis*, three *H. influenzae*, two *S. agalactiae*, two *L. monocytogenes*, and one *C. neoformans/gattii* target detection (Fig. 1B). Among patients with viral detections, HSV-1, HSV-2, HHV-6, and VZV were found predominantly in adults, whereas EV and HPeV were significantly more common in pediatric patients (*P* < 0.01; Fig. 2A). Most bacterial detections also occurred in adults, except for *N. meningitidis*, for which two of three detections occurred in children (Fig. 2B).

### Agreement among syndromic multiplex panel testing platforms

Complete agreement was observed between FilmArray and QIAstat-Dx for HSV-2, *H. influenzae*, *N. meningitidis*, *S. agalactiae*, and *C. neoformans/gattii* panel analytes. Across the remaining viral targets, PPA ranged from 0.67 to 0.93 and NPA from 0.98 to 1.00, with κ values of 0.80–0.92 (Table 2). EV showed the greatest discordance, with 70 FilmArray-positive and 60 QIAstat-Dx-positive target detections, yielding a PPA of 0.86 and an NPA of 1.00. For the remaining bacterial targets, PPA was 0.89 for *S. pneumoniae* and 0.50 for *L. monocytogenes*, with NPA of 1.00 for both.

**TABLE 2:** Inter-platform agreement for shared target detections between FilmArray ME and QIAstat-Dx ME panels in clinical cerebrospinal fluid specimens^*a*^

	Pathogen	F+/Q+	F−/Q+	F−/Q−	F+/Q−	Overall agreement	Cohen’s κ score	PPA (95% CI)	NPA (95% CI)
Viruses	Enterovirus	60	0	112	10	0.95	0.88	0.86 (0.75–0.93)	1.00 (0.96–1.00)
	Herpes simplex virus 1	12	1	168	1	0.99	0.92	0.92 (0.62–1.00)	0.99 (0.96–1.00)
	Herpes simplex virus 2	8	0	174	0	1.00	1.00	1.00 (0.60–1.00)	1.00 (0.97–1.00)
	Human herpesvirus 6	10	3	168	1	0.98	0.82	0.91 (0.57–1.00)	0.98 (0.95–1.00)
	Human parechovirus	4	0	176	2	0.99	0.80	0.67 (0.24–0.94)	1.00 (0.97–1.00)
	Varicella zoster virus	39	2	138	3	0.97	0.92	0.93 (0.79–0.98)	0.99 (0.94–1.00)
Bacteria	*Escherichia coli K1*	0	0	182	0	1.00	na	na	1.00 (0.97–1.00)
	*Haemophilus influenzae*	3	0	179	0	1.00	1.00	1.00 (0.31–1.00)	1.00 (0.97–1.00)
	*Listeria monocytogenes*	1	0	180	1	0.99	0.66	0.50 (0.03–0.97)	1.00 (0.97–1.00)
	*Neisseria meningitidis*	3	0	179	0	1.00	1.00	1.00 (0.31–1.00)	1.00 (0.97–1.00)
	*Streptococcus agalactiae*	2	0	180	0	1.00	1.00	1.00 (0.20–1.00)	1.00 (0.97–1.00)
	*Streptococcus pneumoniae*	8	0	173	1	0.99	0.94	0.89 (0.51–0.99)	1.00 (0.97–1.00)
Fungi	*Cryptococcus neoformans/gattii*	1	0	181	0	1.00	1.00	1.00 (0.05–1.00)	1.00 (0.97–1.00)

^*a*^ Abbreviations: F, FilmArray ME; Q, QIAstat-Dx ME; PPA, positive percent agreement; NPA, negative percent agreement. Cell counts are shown as inter-assay agreement categories using FilmArray ME as the non-reference comparator: F+/Q+, positive by both assays; F−/Q+, positive by QIAstat-Dx ME only; F−/Q−, negative by both assays; F+/Q−, positive by FilmArray ME only. PPA was calculated as F+/Q+ / (F+/Q+ + F+/Q−), and NPA as F−/Q− / (F−/Q− + F−/Q+). na, not estimable because no FilmArray-positive specimens were available.

### Adjudication of discordant test results

Target-specific LDT testing showed that discordant results were concentrated among low-level viral target detections. Among the 10 FilmArray-positive/QIAstat-Dx-negative EV detections, four were confirmed by LDT with estimated target levels below the LDT LLoQ (100, 100, 100, and 700 copies/mL; Table S4). For HSV-1, both the single FilmArray-positive/QIAstat-Dx-negative detection and the single FilmArray-negative/QIAstat-Dx-positive detection were confirmed by LDT, each with an estimated target level below the LLoQ (100 copies/mL). For HHV-6, the single FilmArray-positive/QIAstat-Dx-negative detection and all three FilmArray-negative/QIAstat-Dx-positive detections were confirmed by LDT below the LLoQ (100, 100, 100, and 200 copies/mL). For VZV, 1 of 3 FilmArray-positive/QIAstat-Dx-negative detections and both FilmArray-negative/QIAstat-Dx-positive discordant target detections were confirmed by LDT below the LLoQ (100, 100, and 300 copies/mL). In contrast, neither of the two discordant HPeV target detections were confirmed by LDT. Likewise, the FilmArray-only *L. monocytogenes* and *S. pneumoniae* target detections were not detected by the corresponding LDT (Table S4). Taken together, these adjudication results indicate that inter-platform discordance was concentrated among low-level viral detections, particularly for EV, while a subset of FilmArray-only viral and bacterial target detections remained negative by the corresponding LDT.

### Impact of pathogen load on inter-platform discordance

**Fig 3:**
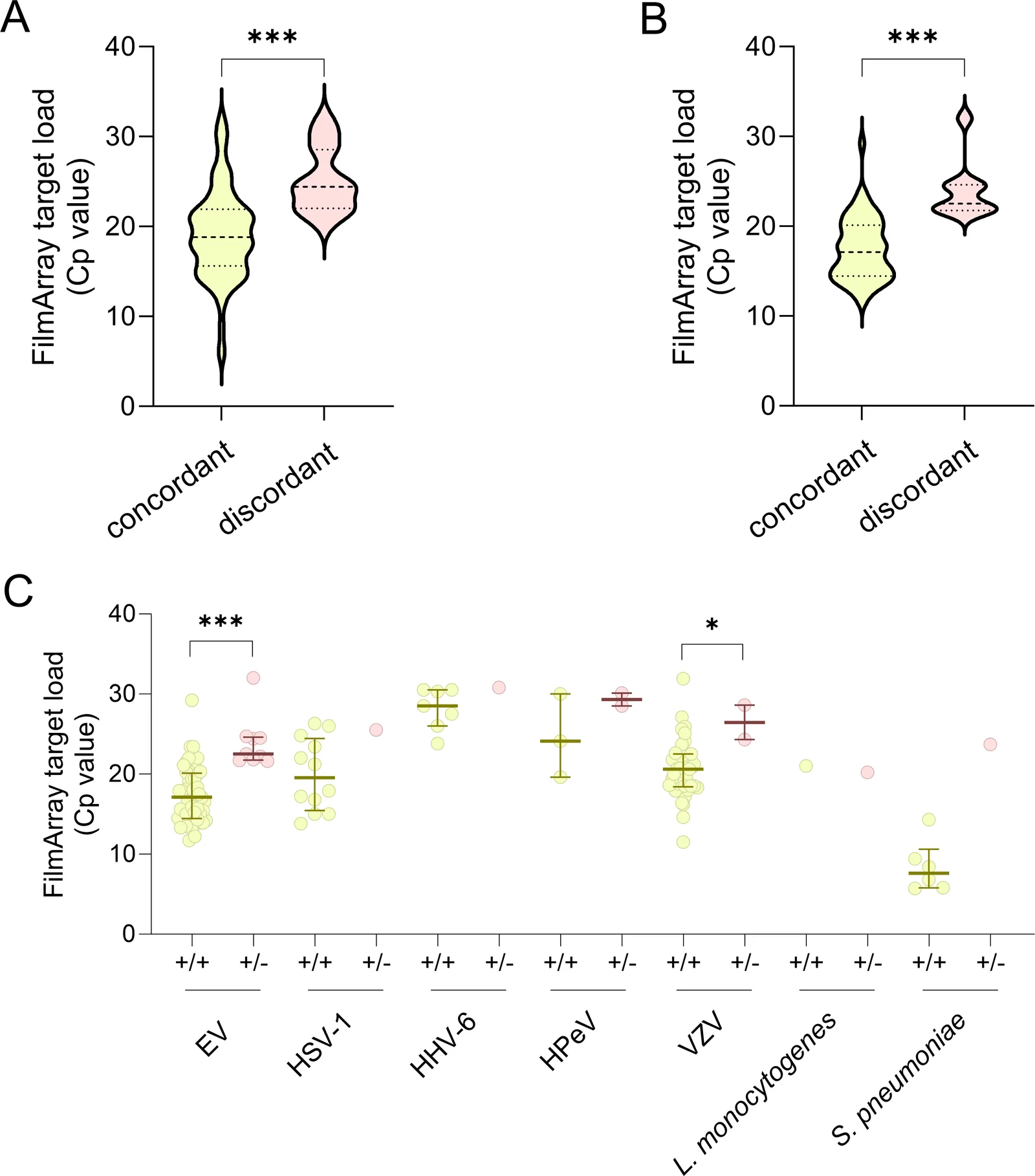
BioFire FilmArray Cp values in concordant-positive and FilmArray-only positive target detections from clinical cerebrospinal fluid specimens. BioFire FilmArray Cp values are shown for concordant-positive FilmArray+/QIAstat-Dx+ (+/+) and FilmArray-only positive (+/−) target detections from clinical cerebrospinal fluid specimens, shown for all positive target detections combined (**A**), enterovirus-positive target detections (**B**), and individual panel analytes (**C**). Horizontal bars indicate medians, and whiskers indicate the 25th and 75th percentiles. *P* values were calculated using the Mann-Whitney *U* test; *P < 0.05 and ***P < 0.001. Cp, crossing point; EV, enterovirus; HSV-1, herpes simplex virus 1; HHV-6, human herpesvirus 6; HPeV, human parechovirus; VZV, varicella-zoster virus; *L. monocytogenes*, *Listeria monocytogenes*; *S. pneumoniae*, *Streptococcus pneumoniae*.

To assess the relationship between target load and inter-platform discordance, Ct values from QIAstat-Dx and Cp values from FilmArray were compared. Across assessed analyte targets, QIAstat-Dx Ct values were higher in QIAstat-Dx-only positive target detections than in concordant-positive target detections, consistent with lower target burden in discordant detections. This difference reached significance for VZV and showed the same trend for HSV-1 and HHV-6 (Fig. S2). Likewise, the median FilmArray Cp value was significantly higher in FilmArray-only positive target detections than in concordant-positive target detections (24.4 [IQR, 22.2–28.6] versus 18.8 [IQR, 15.70–21.98]; *P* < 0.001; Fig. 3A). A similar association was observed within the EV subgroup and across other viral and bacterial analytes, further supporting lower target burden in discordant target detections (Fig. 3B and C).

### Additional performance assessment using dilution experiments and EQA specimens

To further assess the analytical performance of FilmArray and QIAstat-Dx ME panels, serial dilution experiments were performed in triplicate for HSV-1, HSV-2, and different EV types, including coxsackievirus B1, coxsackievirus B6, and echovirus E9, at concentrations of 100, 1,000, and 10,000 copies/mL (Fig. S3). Across these dilution experiments, FilmArray and QIAstat-Dx showed broadly comparable detection rates for the tested analytes.

Analysis of 10 EQA specimens likewise showed concordant results between the two platforms for HSV-1, HSV-2, HPeV, and VZV. In contrast, EV-related differences were again observed, with FilmArray detecting five EV-positive EQA specimens, whereas QIAstat-Dx detected three (Table S5). The discordant FilmArray-only EV detections were also detected by the corresponding LDT, in line with the clinical cohort findings showing that FilmArray more frequently detected low-level EV-positive specimens than QIAstat-Dx.

### Characterization of EV molecular diversity

Because EV showed the greatest inter-platform discordance between FilmArray and QIAstat-Dx, molecular typing was performed to assess whether these differences were primarily related to low target abundance or might also reflect differences in EV-type coverage and inclusivity of the two multiplex panel assays. EV molecular typing yielded analyzable sequences in 68 EV-positive specimens, including three EQA materials. Multiple EV types were identified, with echoviruses (*n* = 45) being the predominant type detected (Table 3). Among the 68 EV-typed specimens, 58 showed EV detection by both platforms, whereas 10 were positive only by FilmArray. The distribution of EV types overlapped between these groups, with no clear type-specific segregation. Several EV types, including echovirus 6, echovirus 18, EV C96, coxsackievirus B5, and coxsackievirus B3, were detected in both groups, arguing against a major EV-type-specific detection bias. Instead, these findings suggest that most inter-platform discordances were primarily driven by low target abundance rather than by major differences in EV-type coverage or multiplex panel inclusivity.

**TABLE 3:** Enterovirus types identified in cerebrospinal fluid specimens with concordant and discordant detection by FilmArray ME and QIAstat-Dx ME panel testing^*a*^

Patient ID	Age (years)	Gender	Discordance pattern	Enterovirus species	Enterovirus type
1	3	F	F+/Q+	A	Coxsackievirus A16
2	3	F	F+/Q+	B	Coxsackievirus A9
3	2	M	F+/Q+	B	Coxsackievirus A9
4	2	F	F+/Q+	B	Coxsackievirus A9
5	3	M	F+/Q+	B	Coxsackievirus B5
6	19	M	F+/Q+	B	Coxsackievirus B5
7	1	M	F+/Q+	B	Coxsackievirus B5
8	34	M	F+/Q+	B	Coxsackievirus B5
9	2	M	F+/Q+	B	Echovirus E11
10	2	F	F+/Q+	B	Echovirus E11
11	26	F	F+/Q+	B	Echovirus E13
12	2	M	F+/Q+	B	Echovirus E18
13	2	M	F+/Q+	B	Echovirus E18
14	2	F	F+/Q+	B	Echovirus E18
15	2	M	F+/Q+	B	Echovirus E18
16	51	M	F+/Q+	B	Echovirus E18
17	<1	M	F+/Q+	B	Echovirus E18
18	23	M	F+/Q+	B	Echovirus E18
19	22	M	F+/Q+	B	Echovirus E18
20	46	M	F+/Q+	B	Echovirus E30
21	3	F	F+/Q+	B	Echovirus E6
22	38	F	F+/Q+	B	Echovirus E6
23	37	F	F+/Q+	B	Echovirus E6
24	49	M	F+/Q+	B	Echovirus E6
25	2	F	F+/Q+	B	Echovirus E6
26	32	F	F+/Q+	B	Echovirus E7
27	29	F	F+/Q+	B	Echovirus E9
28	5	M	F+/Q+	B	Echovirus E9
29	35	F	F+/Q+	B	Echovirus E9
30	9	F	F+/Q+	B	Echovirus E9
31	29	F	F+/Q+	B	Echovirus E9
32	35	F	F+/Q+	B	Echovirus E9
33	<1	M	F+/Q+	B	Echovirus E9
34	7	M	F+/Q+	B	Echovirus E9
35	1	M	F+/Q+	B	Echovirus E9
36	7	F	F+/Q+	B	Echovirus E9
37	13	M	F+/Q+	B	Echovirus E9
38	5	F	F+/Q+	B	Echovirus E9
39	8	M	F+/Q+	B	Echovirus E9
40	7	M	F+/Q+	B	Echovirus E9
41	9	M	F+/Q+	B	Echovirus E9
42	<1	M	F+/Q+	B	Echovirus E9
43	6	F	F+/Q+	B	Echovirus E9
44	<1	M	F+/Q+	B	Echovirus E9
45	16	F	F+/Q+	B	Echovirus E9
46	<1	M	F+/Q+	B	Echovirus E9
47	8	M	F+/Q+	B	Echovirus E9
48	4	F	F+/Q+	B	Echovirus E9
49	26	F	F+/Q+	B	Enterovirus B88
50	36	F	F+/Q+	C	Enterovirus C96
51	62	F	F+/Q+	C	Enterovirus C96
52	39	F	F+/Q+	C	Enterovirus C96
53	36	F	F+/Q+	C	Enterovirus C96
54	34	M	F+/Q+	C	Enterovirus C96
55	32	M	F+/Q+	C	Enterovirus C96
56	39	F	F+/Q+	C	Enterovirus C96
57	5	M	F+/Q+	C	Enterovirus C96
58	<1	M	F+/Q-	B	Coxsackievirus B3
59	1	M	F+/Q−	B	Coxsackievirus B5
60	63	F	F+/Q−	B	Echovirus E18
61	28	F	F+/Q−	B	Echovirus E30
62	27	F	F+/Q−	B	Echovirus E4
63	28	M	F+/Q−	B	Echovirus E6
64	<1	M	F+/Q−	B	Enterovirus B
65	25	F	F+/Q−	C	Enterovirus C96
66 (EQA)	NA	NA	F+/Q+	B	Coxsackievirus B3
67 (EQA)	NA	NA	F+/Q−	B	Echovirus E30
68 (EQA)	NA	NA	F+/Q−	C	Coxsackievirus A24

^*a*^ F+/Q+, positive by both FilmArray ME and QIAstat-Dx ME; F+/Q−, positive by FilmArray ME and negative by QIAstat-Dx ME. EQA, external quality assessment; NA, not applicable. Enterovirus typing was performed on enterovirus-positive CSF specimens and EQA materials with analyzable sequence data.

## DISCUSSION

In this retrospective multicenter comparison, FilmArray and QIAstat-Dx ME showed high agreement across shared CSF targets, with complete concordance for HSV-2, *H. influenzae*, *N. meningitidis*, *S. agalactiae*, and *C. neoformans/gattii*. This overall pattern is consistent with previous comparative studies reporting high agreement and broadly comparable clinical performance of QIAstat-Dx in routine meningoencephalitis diagnostics (1, 5, 6). Building on these studies, the present analysis adds quantitative LDT-based discrepancy resolution and EV molecular characterization, permitting a more direct assessment of residual inter-platform discordance in relation to target burden and viral diversity. Inter-platform discordance was infrequent overall and concentrated in low-level viral detections, indicating that the principal performance differences emerged near the analytical limit of detection. The lowest PPA values were observed for EV (86%) and HPeV (67%), with NPA remaining high (0.98–1.00) across these analytes.

For EV, QIAstat-Dx yielded fewer positive results than FilmArray, and LDT-positive discrepant clinical specimens showed estimated below-LLoQ target levels of 100–700 copies/mL. Higher FilmArray Cp values in discordant than in concordant EV-positive specimens further support that these additional detections clustered at the lower limit of reliable detection. A similar pattern was observed in EQA material, where two FilmArray-only EV detections were also detected by the corresponding LDT. Because FilmArray served as the comparator assay and LDT-based adjudication was restricted to discordant specimens, these results should be interpreted as discrepancy-resolved agreement data rather than formal evidence of assay equivalence or reference-standard-based diagnostic accuracy. Within those constraints, residual discordance was infrequent and largely confined to low-burden viral detections.

The spike-in experiments provided additional context for interpreting the EV discordances. No inter-platform difference was observed when cultured EV strains were diluted in negative CSF, arguing against a uniform intrinsic deficit of QIAstat-Dx under controlled conditions. The discordance observed in native clinical specimens is therefore more consistent with specimen-related effects not reproduced by spike-in models. Erythrocyte and hemoglobin contamination can inhibit EV-specific RT-PCR in CSF (13), and short-term storage of native CSF has been associated with RNA target loss, most pronounced at room temperature, which may impair RNA integrity and compromise borderline detections (14). Collectively, these data support a specimen-dependent explanation for discordant EV detection rather than a consistent EV-type-dependent analytical limitation. However, assay-related factors may still contribute at low copy number, particularly differences in multiplex chemistry, inclusivity across EV-type diversity, and primer/probe performance against variant strains (5, 15, 16). The EV-typing data further support this interpretation. Consistent with prior EV surveillance studies (17), the present cohort was dominated by EV-B, although with a distinct distribution characterized by echovirus 9 as the most frequent type, followed by echovirus 18, EV-C96, echovirus 6, and coxsackievirus B5. Rare detections also included EV B88, which has been implicated in acute flaccid paralysis (18). No evident EV-type segregation was identified between concordant specimens and FilmArray-only detections, and the typing results showed a broad spectrum of EV-A, EV-B, and EV-C species. Since EV-type-dependent differences in CSF viral load have been described in EV-associated CNS infections, and EV sequence diversity can affect low copy number PCR detection, the most frugal interpretation is an interaction between low viral burden and EV diversity rather than a narrow EV-type-restricted detection gap in QIAstat-Dx (7, 19).

Published comparative data place these findings in context. A meta-analysis of the BioFire FilmArray ME panel reported pooled sensitivity and specificity of 90% and 97%, respectively (20). A recent review synthesizing seven clinical evaluations of the QIAstat-Dx ME panel likewise found generally high agreement, with only limited variation in head-to-head studies with FilmArray (21). Adjudicated false positive results were concentrated in *S. pneumoniae* and *S. agalactiae*, while false negative results were seen mainly with HSV-1, HSV-2, EV, and *C. neoformans/gattii* (20). Direct comparisons of QIAstat-Dx reported PPA values of 92.5%–100% and NPA values of 94.1%–99.9% across shared targets (1, 5, 6). Reduced QIAstat-Dx detection near the limit of detection has been documented for low-level positive HSV-1/HSV-2 specimens and for selected EV, *H. influenzae*, and *S. pneumoniae* targets in clinical comparisons (5, 6). Conversely, some studies reported fewer unconfirmed-positive results with QIAstat-Dx than with FilmArray (6).

The present data align with these findings and further show that discordance was bidirectional. QIAstat-Dx-only detections of HSV-1, HHV-6, and VZV were detected by target-specific LDT testing, whereas some FilmArray-only detections of HPeV, *L. monocytogenes*, and *S. pneumoniae* were not detected by the corresponding LDT. However, the target-specific LDTs used for adjudication had an LLoQ of 1,000 copies/mL, representing the lowest concentration at which quantitative results could be reported with acceptable accuracy and precision. Samples with target levels below this threshold could still be detected by the assay but were reported as positive without reliable quantification. Therefore, non-confirmation of a panel-positive result cannot be equated with false positivity. Within these constraints, the present data, in line with the available literature, suggest that FilmArray may detect additional low-level signals for selected targets, whereas QIAstat-Dx may yield fewer unconfirmed positives.

Neither platform appeared uniformly superior across all targets; rather, the principal difference concerned the handling of borderline-positive specimens rather than in overall diagnostic performance across the ME panel. These near-threshold differences may still be clinically relevant because they affect diagnostic certainty and downstream management of suspected CNS infections. In HSV encephalitis, although acyclovir should be initiated on clinical suspicion, an initially negative CSF PCR may reduce diagnostic confidence, delay repeat sampling or continuation of antiviral treatment, and has been associated with worse neurologic outcome (22, 23). For EV and HPeV, missed detection does not usually delay pathogen-specific therapy, but it may still have important clinical consequences, particularly in infants and young children, in whom a virologic diagnosis can support antimicrobial stewardship, shorten hospitalization, and enable earlier discontinuation of empirical antibiotics (24, 25). By contrast, failure to identify bacterial or cryptococcal meningitis is of more immediate consequence because negative syndromic panel results do not reliably exclude all bacterial pathogens, including *L. monocytogenes*, and may also miss cryptococcal disease when clinical suspicion persists (26–30). Conversely, false positive results may also affect clinical management by prompting unnecessary antimicrobial therapy and, in the case of an erroneous meningococcal result, avoidable isolation measures and chemoprophylaxis for close contacts (31, 32).

This is also highly relevant for HHV-6, where a positive CSF result only rarely reflects true HHV-6 encephalitis and may instead represent chromosomally integrated HHV-6 or incidental reactivation (33, 34). Similar interpretive constraints apply to EV and HPeV. In neonates and young infants, particularly in sepsis-like presentations, low-level CSF positivity may occur without pleocytosis, and plasma-CSF viral load comparisons suggest that some such detections may reflect passive transfer from blood rather than intrathecal replication when plasma viral loads markedly exceed those in CSF (35). This likewise applies for HPeV, although the absence of pleocytosis does not exclude enteroviral CNS infection in young infants (35–37). More generally, the positive predictive value of syndromic CSF panels declines in low-pretest-probability specimens, particularly when CSF pleocytosis or protein elevation is absent, and diagnostic-stewardship strategies restricting testing in such samples may improve yield and reduce false-positive reporting (38–40). Accordingly, at very low copy number, inter-platform differences in analytical sensitivity may influence not only detection rates but also clinical specificity and post-test interpretation.

In this setting, semi-quantification of pathogen burden provided by QIAstat-Dx may support interpretation of low-level detections. In the present cohort, discordant QIAstat-Dx-positive specimens had higher Ct values than concordant positives; likewise most missed detections in previous comparative studies occurred at high Ct values (>35) (5, 6). Recent data further suggest that CSF multiplex PCR Ct values may carry prognostic or risk-stratification value in community-acquired meningoencephalitis (41). However, Ct values should be regarded as assay-specific semiquantitative adjuncts rather than interchangeable measures of absolute pathogen burden and may be most useful for identifying results that merit additional target-specific testing or closer clinicopathologic correlation. In the uncommon setting of multiple panel detections, the semiquantitative output of QIAstat-Dx may also provide additional interpretive context regarding the relative burden of co-detected targets and thereby support assessment of the most likely clinically relevant pathogen, although this potential advantage was not specifically evaluated in the present cohort. From a laboratory implementation perspective, both platforms provide turnaround times compatible with acute CNS infection management. The practical distinction is therefore driven mainly by target composition. The QIAstat-Dx version evaluated here did not include CMV, necessitating access to separate CMV PCR testing when clinically indicated, particularly in immunocompromised patients (1, 42–45). Conversely, QIAstat-Dx includes *S. pyogenes* and *M. pneumoniae*, targets not detected in the present series but potentially relevant in selected settings, including pediatric encephalitis. Platform selection should therefore be aligned with local patient populations, anticipated pathogen spectrum, and availability of supplementary PCR assays for targets absent from the chosen panel.

This study has some limitations. Positive numbers were small for some pathogens, particularly *C. neoformans/gattii*, and the less frequent bacterial targets, limiting robust target-specific performance estimates. The study design was retrospective, and discordant analyses relied on residual specimen availability and adjudication by pathogen-specific LDT rather than on a fully independent reference standard applied to all samples. Because LDT-based adjudication was restricted to discordant specimens, the discrepancy-resolved analysis should be interpreted as adjudicated rather than unbiased diagnostic accuracy. Finally, syndromic panels interrogate only predefined targets, so infections caused by organisms outside the panel remain undetectable without additional testing.

In conclusion, BioFire FilmArray and QIAstat-Dx showed high agreement across shared CSF meningitis/encephalitis panel targets. Inter-platform discordance was uncommon and concentrated in low-burden viral detections, particularly EV, where FilmArray more often identified specimens near the limit of detection. Adjudication, dilution experiments, and molecular typing suggest that these differences were driven primarily by low pathogen burden, with EV-type diversity contributing only in combination with near-threshold viral loads rather than through a consistent EV-type-specific detection gap. These findings refine interpretation of discordant syndromic CSF panel results and underscore that borderline viral positives are best interpreted within a laboratory-based diagnostic framework that integrates supplementary target-specific testing, clinical context, and, where available, semiquantitative signal characteristics such as Ct values.

## Data Availability

The raw Illumina sequencing reads and associated sample records generated in this study have been submitted to the European Nucleotide Archive under project accession PRJEB122085, sample accessions ERS30888483–ERS30888547, and read accessions ERR17623852–ERR17623856, ERR17624074–ERR17624133.

## SUPPLEMENTAL MATERIAL

Fig. S1 spectrum.01333-26-s0001.pdf Clinical cerebrospinal fluid specimens from four participating centers and external quality assessment (EQA) specimens included in the study. Primary inter-platform agreement analyses were performed on the 182 clinical specimens; EQA specimens were analyzed separately.

Fig. S2 spectrum.01333-26-s0002.pdf QIAstat-Dx cycle threshold values in concordant-positive and QIAstat-Dx-only positive target detections from clinical cerebrospinal fluid specimens.

Fig. S3 spectrum.01333-26-s0003.pdf Detection rates of FilmArray and QIAstat-Dx in serial dilution experiments.

Table S1 spectrum.01333-26-s0004.docx Viral, bacterial, and fungal targets detected by the BioFire FilmArray and the QIAstat-Dx ME panel (version 1.1).

Table S2 spectrum.01333-26-s0005.docx Demographic characteristics of the clinical cohort.

Table S3 spectrum.01333-26-s0006.docx Characteristics of pathogen-specific laboratory developed tests used for discrepancy adjudication.

Table S4 spectrum.01333-26-s0007.docx Laboratory-developed test adjudication of discordant FilmArray and QIAstat-Dx ME panel results in clinical cerebrospinal fluid specimens.

Table S5 spectrum.01333-26-s0008.docx Laboratory developed test adjudication of external quality assessment specimens tested by FilmArray and QIAstat-Dx ME panels.

ASM does not own the copyrights to Supplemental Material that may be linked to, or accessed through, an article. The authors have granted ASM a non-exclusive, world-wide license to publish the Supplemental Material files. Please contact the corresponding author directly for reuse.
