# Supplementary material for: Quantitative and molecular adjudication of BioFire FilmArray and QIAstat-Dx discordance in meningitis/encephalitis diagnostics
Source: Microbiol Spectr. 2026 Sep 2;14(10):e01333-26. doi: 10.1128/spectrum.01333-26 (PMC13689469; doi:10.1128/spectrum.01333-26)
Supplement: Supplementary file 1 [file spectrum.01333-26-s0001.pdf]

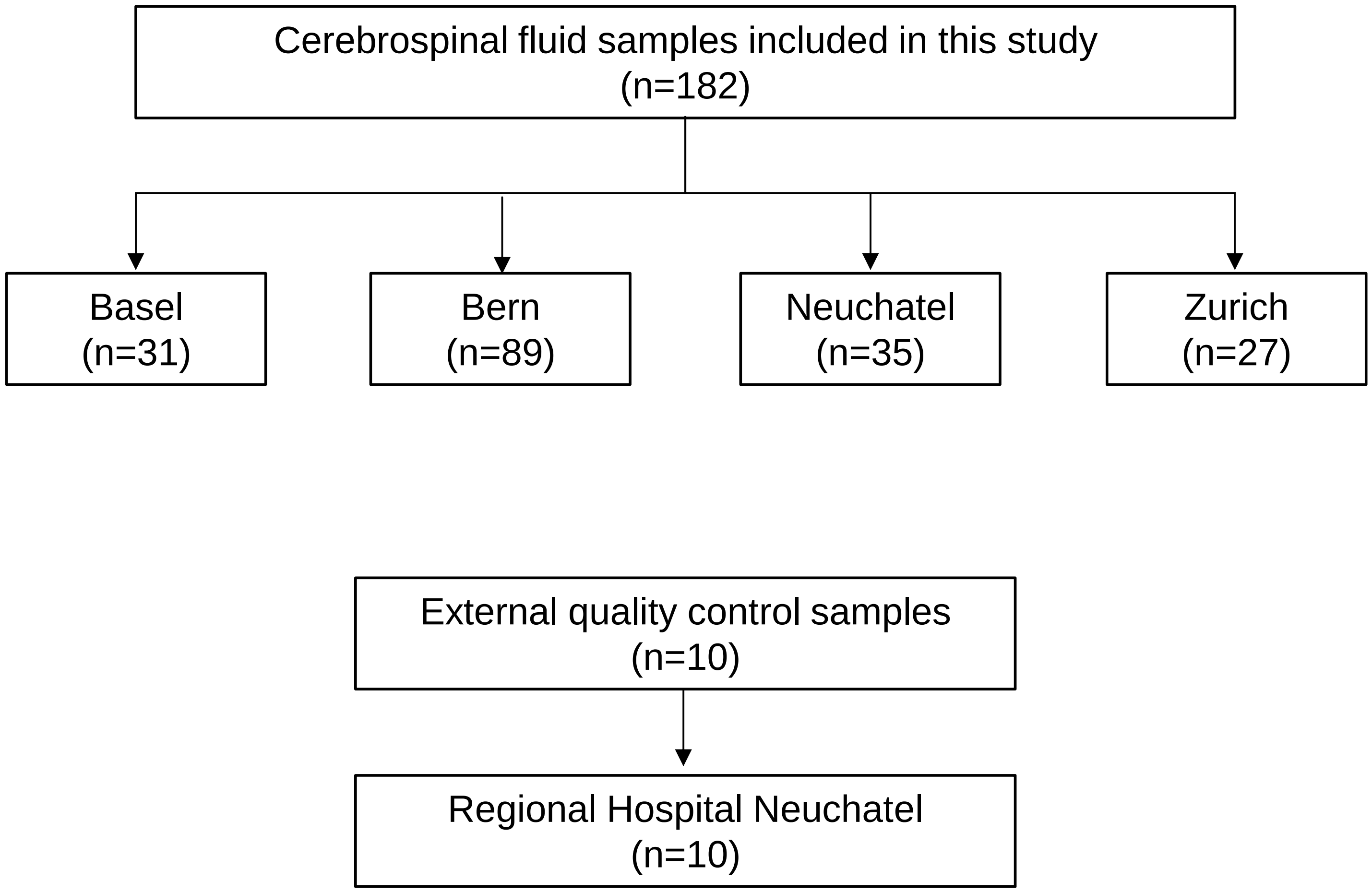

Supplementary Figure 1. Clinical cerebrospinal fluid specimens from four participating centers and external quality assessment (EQA) specimens included in the study. Primary inter-platform agreement analyses were performed on the 182 clinical specimens; EQA specimens were analyzed separately.
