# Supplementary material for: Quantitative and molecular adjudication of BioFire FilmArray and QIAstat-Dx discordance in meningitis/encephalitis diagnostics
Source: Microbiol Spectr. 2026 Sep 2;14(10):e01333-26. doi: 10.1128/spectrum.01333-26 (PMC13689469; doi:10.1128/spectrum.01333-26)
Supplement: Supplementary file 2 [file spectrum.01333-26-s0002.pdf]

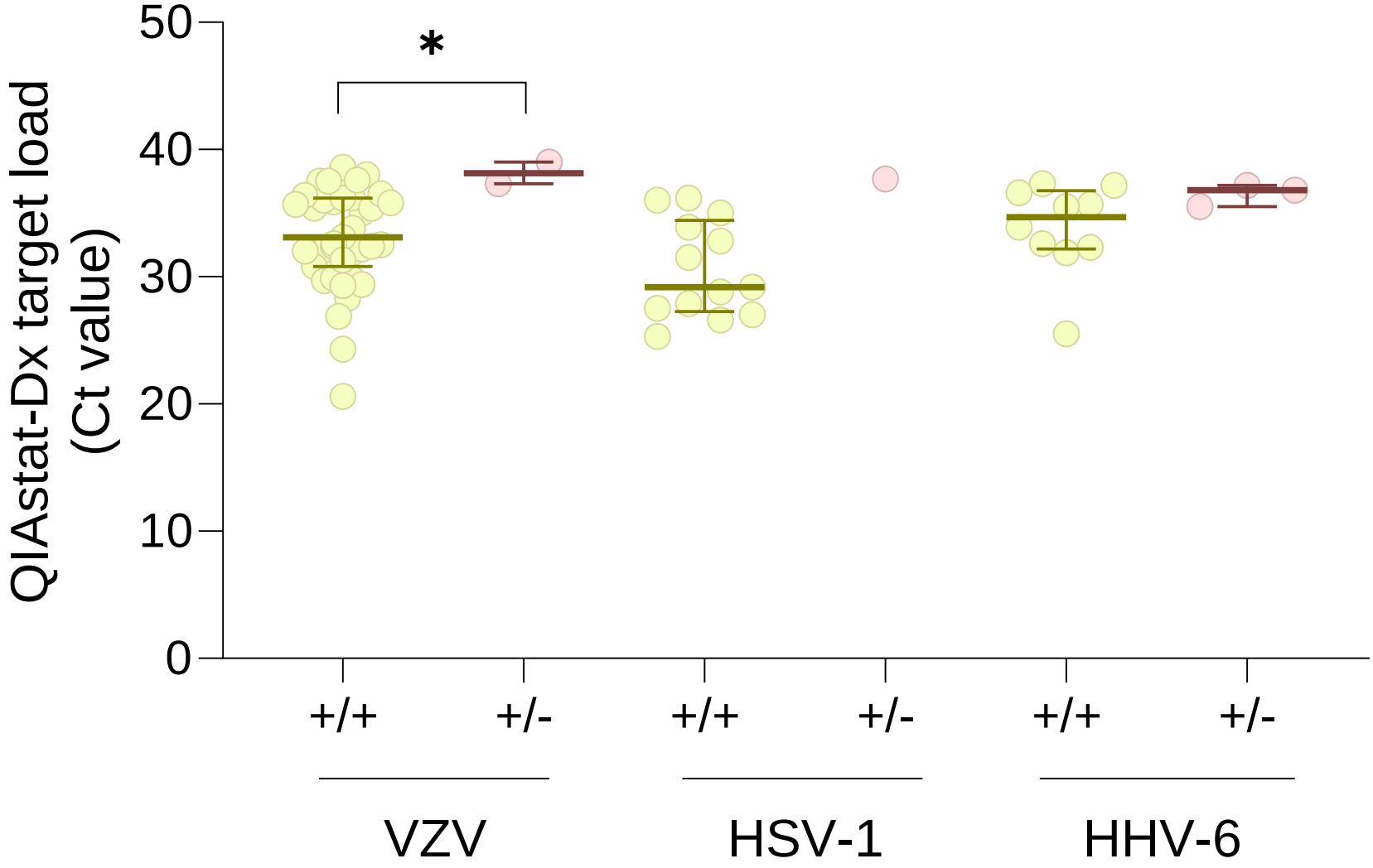

Supplementary Figure 2. QIAstat-Dx cycle threshold values in concordant-positive and QIAstat-Dx-only positive target detections from clinical cerebrospinal fluid specimens. QIAstat-Dx cycle threshold (Ct) values are shown for concordant-positive QIAstat-Dx+/FilmArray+ (+/+) target detections and QIAstat-Dx-only positive (+/−) target detections. Horizontal bars indicate medians, and whiskers indicate the 25th and 75th percentiles. P values were calculated using the Mann–Whitney U test; * $p < 0.05$. Abbreviations: Ct, cycle threshold; HSV-1, herpes simplex virus 1; HHV-6, human herpesvirus 6; VZV, varicella-zoster virus.
