# Supplementary material for: Quantitative and molecular adjudication of BioFire FilmArray and QIAstat-Dx discordance in meningitis/encephalitis diagnostics
Source: Microbiol Spectr. 2026 Sep 2;14(10):e01333-26. doi: 10.1128/spectrum.01333-26 (PMC13689469; doi:10.1128/spectrum.01333-26)
Supplement: Supplementary file 3 [file spectrum.01333-26-s0003.pdf]

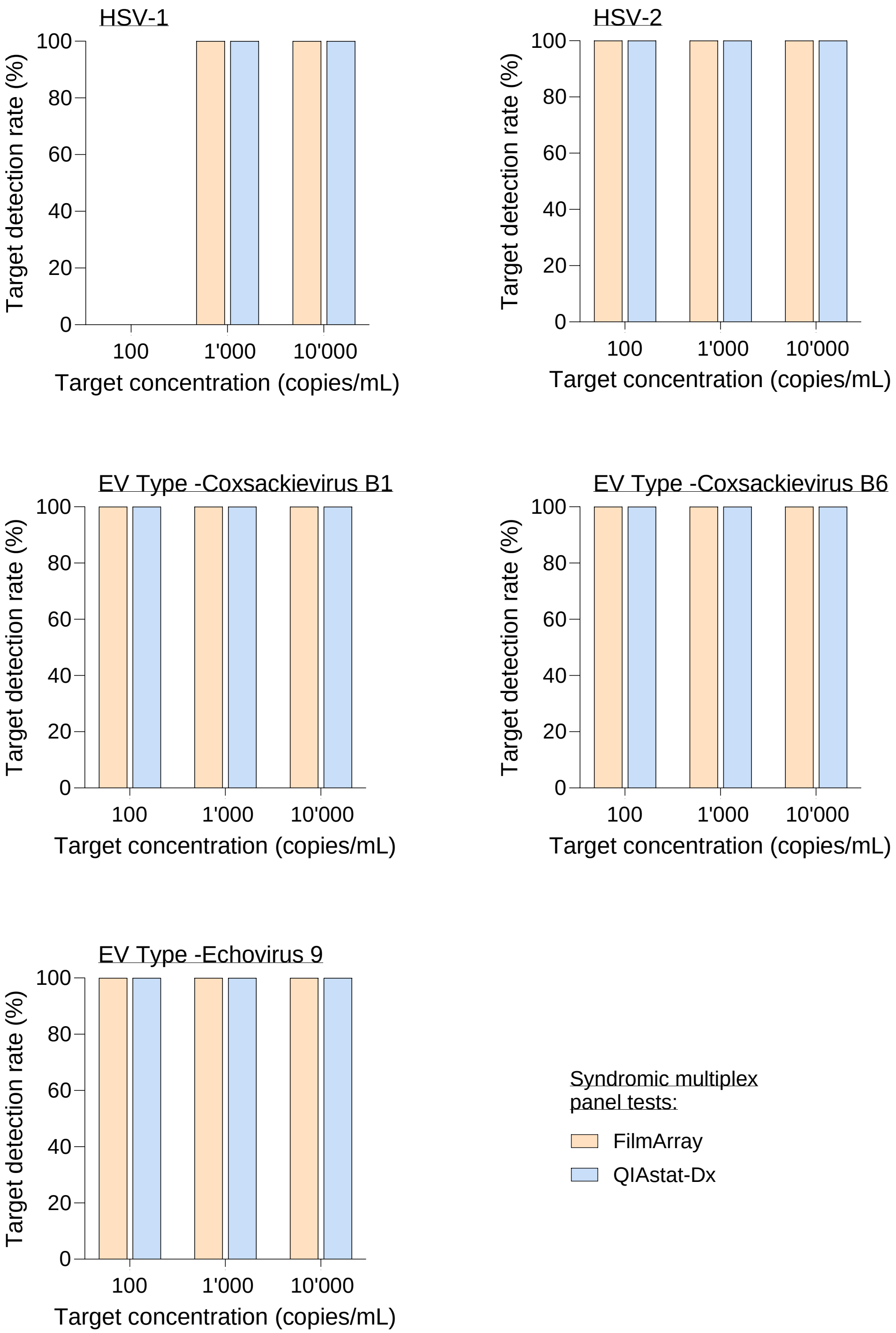

Supplementary Figure 3. Detection rates of FilmArray and QIAstat-Dx in serial dilution experiments.

Negative CSF specimens were spiked with high-titer cell-culture supernatants containing whole virus particles of HSV-1, HSV-2, and selected enterovirus types, including coxsackievirus B1, coxsackievirus B6, and echovirus 9. The resulting high-titer CSF stocks were serially diluted in negative CSF to nominal target concentrations of 100, 1,000, and 10,000 viral genome copies/mL. Target concentrations in the stock preparations and dilution series were verified using the corresponding in-house quantitative LDT calibrated to external plasmid standards. Each dilution was tested in triplicate with FilmArray and QIAstat-Dx. Bars show the proportion of positive detections at each concentration. Abbreviations: HSV-1, herpes simplex virus 1; HSV-2, herpes simplex virus 2; EV, enterovirus.
