# Supplementary material for: Quantitative and molecular adjudication of BioFire FilmArray and QIAstat-Dx discordance in meningitis/encephalitis diagnostics
Source: Microbiol Spectr. 2026 Sep 2;14(10):e01333-26. doi: 10.1128/spectrum.01333-26 (PMC13689469; doi:10.1128/spectrum.01333-26)
Supplement: Supplementary file 4 [file spectrum.01333-26-s0004.docx]

**Supplementary Table 1.** **Viral, bacterial, and fungal targets detected by the BioFire FilmArray and the QIAstat-Dx ME panel (version 1.1).**

|  | **BioFire FilmArray ME panel** | **QIAstat-Dx ME panel** |
| --- | --- | --- |
| **Viruses** | Enterovirus | Enterovirus |
|  | Cytomegalovirus | – |
|  | Herpes simplex virus 1 | Herpes simplex virus 1 |
|  | Herpes simplex virus 2 | Herpes simplex virus 2 |
|  | Human herpes virus 6 | Human herpes virus 6 |
|  | Human parechovirus | Human parechovirus |
|  | Varicella zoster virus | Varicella zoster virus |
| **Bacteria** | *Escherichia coli K1* | *Escherichia coli K1* |
|  | *Haemophilus influenzae* | *Haemophilus influenzae* |
|  | *Listeria monocytogenes* | *Listeria monocytogenes* |
|  | ̶ | *Mycoplasma pneumoniae* |
|  | *Neisseria meningitidis* | *Neisseria meningitidis* |
|  | *Streptococcus agalactiae* | *Streptococcus agalactiae* |
|  | *Streptococcus pneumoniae* | *Streptococcus pneumoniae* |
|  | *̶* | *Streptococcus pyogenes* |
| **Fungi** | *Cryptococcus neoformans/gattii* | *Cryptococcus neoformans/gattii* |
