# Supplementary material for: Quantitative and molecular adjudication of BioFire FilmArray and QIAstat-Dx discordance in meningitis/encephalitis diagnostics
Source: Microbiol Spectr. 2026 Sep 2;14(10):e01333-26. doi: 10.1128/spectrum.01333-26 (PMC13689469; doi:10.1128/spectrum.01333-26)
Supplement: Supplementary file 5 [file spectrum.01333-26-s0005.docx]

**Supplementary Table 2.** **Demographic characteristics of the clinical cohort**

| Patients (n) | 182 |
| --- | --- |
| Female (n, %) | 83 (45.6%) |
| Children ≤18 years (n, %) | 55 (30.2%) |
| Age (years) | Median: 38  25^th^ P: 8  75^th^ P: 63 |
