# Supplementary material for: Quantitative and molecular adjudication of BioFire FilmArray and QIAstat-Dx discordance in meningitis/encephalitis diagnostics
Source: Microbiol Spectr. 2026 Sep 2;14(10):e01333-26. doi: 10.1128/spectrum.01333-26 (PMC13689469; doi:10.1128/spectrum.01333-26)
Supplement: Supplementary file 6 [file spectrum.01333-26-s0006.docx]

**Supplementary Table 3.** **Characteristics of pathogen-specific laboratory developed tests used for discrepancy adjudication**

| **Pathogen** | **Assay type** | **Target gene/ region** | **Forward primer (5′–3′)** | **Reverse primer (5′–3′)** | **Probe (5′–3′)** | **Final concentration nM** | **Reference** |
| --- | --- | --- | --- | --- | --- | --- | --- |
| Human herpesvirus 6 | qPCR | DNA polymerase | GAAGCAGCAATCGCAACACA | ACAACATGTAACTCGGTGTACGG | AACCCGTGCGCCGCTCCC (FAM-TAMRA) | F 300; R 300;  P 300 | [1] |
| Herpes simplex virus 1 | qPCR | Glycoprotein D | CGGCCGTGTGACACTATCG | CTCGTAAAATGGCCCCTCC | CCATACCGACCACACCGACGAACC (FAM-TAMRA) | F 300; R 300;  P 300 | [2] |
| Herpes simplex virus 2 | qPCR | Glycoprotein G | CGCTCTCGTAAATGCTTCCCT | TCTACCCACAACAGACCCACG | CGCGGAGACATTCGAGTACCAGATCG (VIC-TAMRA) | F 300; R 300;  P 400 | [2] |
| Varicella zoster virus | qPCR | Glycoprotein gB | GTCCCCGTTTTTTGGCCT | GGTGAAAACGATCCATTGCATA | CGGGATGGTGCATACAGAGAACATTCCA  (FAM-TAMRA) | F 300; R 300;  P 200 | Validated in-house LDT |
| Human parechovirus | RT-qPCR | 5′ non-translated region | GTAACASWWGCCTCTGGGSCCAAAAG | GGCCCCWGRTCAGATCCAYAGT | CCTRYGGGTACCTYCWGGGCATCCTTC  (FAM-BHQ1) | F 3300; R 3300;  P 2200 | [3] |
| Enterovirus | RT-qPCR | 5′ non-translated region | GGCCCCTGAATGCGGCTAATCC | GCGATTGTCACCATWAGCAGYCA | CCGACTACTTTGGGWGTCCGTGT (FAM-BHQ1) | F 400; R 400;  P 100 | [4] |
| *Streptococcus pneumoniae* | qPCR | lytA | ACGCAATCTAGCAGATGAAGCA | TCGTGCGTTTAATTCCAGCT | GCCGAAAACGCTTGATACAGGGAG (FAM-BHQ1) | F 900; R 900;  P 200 | [5] |
| *Streptococcus pneumoniae* | qPCR | cspA | GCTGTTTTAGCAGATAGTGAGATCGA | TCCCAGTCGGTGCTGTCA | AATGTTACGCAACTGACGAG (NED-MGB-BHQ1) | F 900; R 900;  P 200 | [6] |

*Streptococcus pneumoniae* LDT: simultaneous detection of lytA and cspA. Abbreviations: qPCR, quantitative PCR; RT-qPCR, reverse-transcription quantitative PCR; LDT, laboratory-developed test; LLoQ, lower limit of quantification. Degenerate bases: R = A/G; S = G/C; W = A/T; Y = C/T.

External calibration standards consisted of plasmids containing the respective pathogen-specific target region. Standards at 10⁶, 10⁴, and 10² copies per mL were included in triplicate in each assay run to generate calibration curves. The LLoQ for adjudication LDTs was 1,000 copies/mL.

**References**

[1]	Watzinger, F., et al., Real-time quantitative PCR assays for detection and monitoring of pathogenic human viruses in immunosuppressed pediatric patients. Journal of clinical microbiology, 2004. 42(11): p. 5189-5198.

[2]	Weidmann, M., U. Meyer-König, and F.T. Hufert, Rapid detection of herpes simplex virus and varicella-zoster virus infections by real-time PCR. Journal of clinical microbiology, 2003. 41(4): p. 1565-1568.

[3]	Nix, W.A., et al., Detection of all known parechoviruses by real-time PCR. Journal of clinical microbiology, 2008. 46(8): p. 2519-2524.

[4]	Gosert, R., U. Heininger, and H.H. Hirsch, Enterovirus detection in patients with acute gastroenteritis in Switzerland. Journal of medical virology, 2018. 90(4): p. 685-691.

[5]	Carvalho, M.d.G.S., et al., Evaluation and improvement of real-time PCR assays targeting lytA, ply, and psaA genes for detection of pneumococcal DNA. Journal of clinical microbiology, 2007. 45(8): p. 2460-2466.

[6]	Park, H.K., H.J. Lee, and W. Kim, Real-time PCR assays for the detection and quantification of Streptococcus pneumoniae. FEMS microbiology letters, 2010. 310(1): p. 48-53.
