# Supplementary material for: Quantitative and molecular adjudication of BioFire FilmArray and QIAstat-Dx discordance in meningitis/encephalitis diagnostics
Source: Microbiol Spectr. 2026 Sep 2;14(10):e01333-26. doi: 10.1128/spectrum.01333-26 (PMC13689469; doi:10.1128/spectrum.01333-26)
Supplement: Supplementary file 7 [file spectrum.01333-26-s0007.docx]

**Supplementary Table 4. Laboratory-developed test adjudication of discordant FilmArray and QIAstat-Dx ME panel results in clinical cerebrospinal fluid specimens**

| **Pathogen** | **Discordance pattern** | **No. of discrepant specimens** | **Target-specific LDT result** | **Target-specific LDT result (copies/mL)** | **Final adjudication** |
| --- | --- | --- | --- | --- | --- |
| Enterovirus | F+/Q− | 10 | 4/10 confirmed | 100, 100,  100, 700 | Partially confirmed positive below LLoQ |
| Herpes simplex virus 1 | F+/Q− | 1 | 1/1 confirmed | 100 | Confirmed positive below LLoQ |
|  | F−/Q+ | 1 | 1/1 confirmed | 100 | Confirmed positive below LLoQ |
| Human herpesvirus 6 | F+/Q− | 1 | 1/1 confirmed | 100 | Confirmed positive below LLoQ |
|  | F−/Q+ | 3 | 3/3 confirmed | 100, 100,  200 | Confirmed positive below LLoQ |
| Human parechovirus | F+/Q− | 2 | 0/2 confirmed | — | Not confirmed |
| Varicella zoster virus | F+/Q− | 3 | 1/3 confirmed | 100 | Partially confirmed positive below LLoQ |
|  | F−/Q+ | 2 | 2/2 confirmed | 100, 300 | Confirmed positive below LLoQ |
| *Listeria monocytogenes* | F+/Q− | 1 | 0/1 confirmed | — | Not confirmed |
| *Streptococcus pneumoniae* | F+/Q− | 1 | 0/1 confirmed | — | Not confirmed |

Abbreviations: F, BioFire FilmArray ME; Q, QIAstat-Dx ME; LDT, laboratory-developed test; LLoQ, lower limit of quantification.

Values below 1,000 copies/mL are below the LDT LLoQ and are shown as estimated target levels only. These results were considered LDT-positive but not reliably quantifiable.

F+/Q− indicates positive by FilmArray ME and negative by QIAstat-Dx ME; F−/Q+ indicates negative by FilmArray ME and positive by QIAstat-Dx ME.
