# Supplementary material for: Quantitative and molecular adjudication of BioFire FilmArray and QIAstat-Dx discordance in meningitis/encephalitis diagnostics
Source: Microbiol Spectr. 2026 Sep 2;14(10):e01333-26. doi: 10.1128/spectrum.01333-26 (PMC13689469; doi:10.1128/spectrum.01333-26)
Supplement: Supplementary file 8 [file spectrum.01333-26-s0008.docx]

**Supplementary Table 5. Laboratory developed test adjudication of external quality assessment specimens tested by FilmArray and QIAstat-Dx ME panels**

| **Pathogen** | **Discordance pattern** | **No. of EQA specimens** | **Target-specific LDT result** | **Target-specific LDT result (copies/mL)** | **Final**  **adjudication** |
| --- | --- | --- | --- | --- | --- |
| Enterovirus | F+/Q+ | 3 | — | — | Concordant  positive |
|  | F+/Q− | 2 | 2/2 confirmed | 100, 300 | Confirmed positive below LLoQ |
| Herpes simplex virus 1 | F+/Q+ | 1 | — | — | Concordant  positive |
| Herpes simplex virus 2 | F+/Q+ | 1 | — | — | Concordant  positive |
| Human parechovirus | F+/Q+ | 2 | — | — | Concordant  positive |
| Varicella-zoster virus | F+/Q+ | 1 | — | — | Concordant  positive |

Abbreviations: F, BioFire FilmArray ME; Q, QIAstat-Dx ME; EQA, external quality assessment; LDT, laboratory-developed test; LLoQ, lower limit of quantification.

Values below 1,000 copies/mL are below the LDT LLoQ and are shown as estimated target levels only. These results were considered LDT-positive but not reliably quantifiable.

F+/Q+ indicates positive by both assays; F+/Q− indicates positive by FilmArray ME and negative by QIAstat-Dx ME.
